# Inferring cellular networks – a review

**DOI:** 10.1186/1471-2105-8-S6-S5

**Published:** 2007-09-27

**Authors:** Florian Markowetz, Rainer Spang

**Affiliations:** 1 Max Planck Institute for Molecular Genetics, Ihnestrasse 63-73, 14195 Berlin, Germany; 2 Princeton University, Lewis-Sigler Institute for Integrative Genomics and Dept. of Computer Science, Princeton, NJ 08544, USA; 3 Present affiliation: University Regensburg, Institute of Functional Genomics, Josef-Engert-Str. 9, 93053 Regensburg, Germany

## Abstract

In this review we give an overview of computational and statistical methods to reconstruct cellular networks. Although this area of research is vast and fast developing, we show that most currently used methods can be organized by a few key concepts. The first part of the review deals with *conditional independence *models including Gaussian graphical models and Bayesian networks. The second part discusses probabilistic and graph-based methods for data from experimental *interventions and perturbations*.

## Introduction

The success of genome sequencing projects has led to the identification of almost all the genes responsible for the biological complexity of several organisms. The next important task is to assign a function to each of these genes. Genes and gene products do not work in isolation; rather, they are connected in highly structured networks of information flow through the cell. The inference of such cellular networks using computational and statistical methods is the topic of this review.

The first two sections describe models based on correlation and statistical dependence using the concept of *conditional independence*. The objective of this kind of modeling is to explain observed correlations between genes by the presence of other genes. The set of models can be ordered according to how deeply correlations are resolved: correlation graphs are the most simple example, and Bayesian networks the most sophisticated.

The last section discusses computational approaches building on observed effects of external interventions into the cell. In modern biology, perturbation experiments are key to inferring gene function and regulatory pathways. A common biological technique is to perturb a gene of interest and to study which other genes' activities are affected. Either, the effects of interventions can be included into conditional independence models, or the observed cause-effect relations can be used directly. We also discuss the situation when instead of direct observations of intervention effects on pathway components, only secondary effects on downstream genes are available.

### Notation

Let *V *be a set of *p *network components. The biological meaning of a "network component" depends on what kind of data we analyze. We mostly speak of network components as genes, since the primary data for inference is microarray data and the network is a transcriptional gene regulatory network. However, the methods are general and can also be applied to protein data [1-3]. In probabilistic models we treat each component *v *∈ *V *as a random variable *X*_*v *_and the set of all components in the model as a random vector ***X ***= (*X*_1_,..., *X*_*p*_). The dataset ***D ***consists of *N *measurements, that is, realizations ***x***^1^,..., ***x***^*N *^of the random vector ***X***. Network components are identified with nodes in a graph. The goal is to find an edge set ℰ
 MathType@MTEF@5@5@+=feaafiart1ev1aaatCvAUfKttLearuWrP9MDH5MBPbIqV92AaeXatLxBI9gBamrtHrhAL1wy0L2yHvtyaeHbnfgDOvwBHrxAJfwnaebbnrfifHhDYfgasaacH8akY=wiFfYdH8Gipec8Eeeu0xXdbba9frFj0=OqFfea0dXdd9vqai=hGuQ8kuc9pgc9s8qqaq=dirpe0xb9q8qiLsFr0=vr0=vr0dc8meaabaqaciaacaGaaeqabaWaaeGaeaaakeaaimaacqWFWesraaa@3785@ representing the dependency structure of the network components. We call the graph *T *= (*V*, ℰ
 MathType@MTEF@5@5@+=feaafiart1ev1aaatCvAUfKttLearuWrP9MDH5MBPbIqV92AaeXatLxBI9gBamrtHrhAL1wy0L2yHvtyaeHbnfgDOvwBHrxAJfwnaebbnrfifHhDYfgasaacH8akY=wiFfYdH8Gipec8Eeeu0xXdbba9frFj0=OqFfea0dXdd9vqai=hGuQ8kuc9pgc9s8qqaq=dirpe0xb9q8qiLsFr0=vr0=vr0dc8meaabaqaciaacaGaaeqabaWaaeGaeaaakeaaimaacqWFWesraaa@3785@) the topology of the cellular network. Depending on the model, *T *can be directed or undirected, cyclic or acyclic. In the important special case, where *T *is a directed acyclic graph (DAG), we call it *G*.

## Conditional independence models

The first section describes statistical models to resolve the correlation structure of genes. The methods can be distinguished by the way they remove influences of other genes from the observed correlations. We first introduce coexpression networks, then discuss models building on statements of full conditional independence or low-order conditional independence.

### Coexpression networks

Biological processes result from the concerted action of interacting molecules. This general observation suggests a simple idea, which has already motivated the first approaches to clustering expression profiles [4,5] and is still widely used in functional genomics. It is called the *guilt-by-association heuristic*: if two genes show similar expression profiles, they are supposed to follow the same regulatory regime. To put it more pointedly: coexpression hints at coregulation. Coexpression networks (also known as relevance networks) are constructed by computing a similarity score for each pair of genes. If similarity is above a certain threshold, the gene pair gets connected in the graph, if not, it remains unconnected. Wolfe *et al*. [6] argue that networks of coexpressed genes provide a widely applicable framework for assigning gene function. They show that coexpression agrees well with functional similarity as it is encoded in the Gene Ontology [7]. Examining coexpression is an integral part of most methods combining diverse data sources to uncover biological relationships between genes or proteins [8,9].

#### Building coexpression networks

The first critical point in building a coexpression network is how to formalize the notion of similarity of expression profiles. Several measures have been proposed, the most simple of which is correlation. In a Gaussian model, zero correlation corresponds to statistical independence. Correlation networks are easy to interpret and can be accurately estimated even if *p *≫ *N*, that is, the number of genes is much larger than the number of samples. Stuart *et al*. [10] have used this approach to build a graph from coexpression across multiple organisms (humans, flies, worms and yeast), finding that many coexpression relationships are conserved over evolution. Correlation networks can be enhanced in several ways. Bickel [11] generalizes them to time series data by introducing a time-lag for correlation. Kostka and Spang [12] introduce the concept of *differential coexpression*, which can be interpreted as the gain or loss of a regulatory mechanism. They formulate a method to find sets of genes, which are highly correlated under one condition (e.g. in healthy cells), but show random behavior under a second condition (e.g. in tumor cells).

Correlation is a linear measure of independence, non-linear dependencies between genes are not necessarily found. This problem can be addressed by using networks built from more flexible similarity measures like pair-wise mutual information [13], or non-linear kernel-functions [14]. Yamanishi *et al*. [1] use kernel functions for supervised network reconstruction by tuning kernel parameters in known parts of a protein-interaction graph and then using them to infer unknown parts. Kato *et al*. [2] weight different data sources according to noise and information content and combine them into a single kernel function.

The second critical step in building coexpression networks is assessing the significance of results. Many pairs of genes show similar behavior in expression profiles by chance even though they are not biologically related. A practical, though time-consuming strategy consists of permuting the data matrix and comparing the network obtained from real data with the distribution of similarity scores achieved in the permutations, as Bickel [11] does to estimate the false discovery rate of spurious connections. In the supervised setting of Yamanishi *et al*. [1] cross-validation can be applied to choose optimal parameters.

#### Problems of coexpression based approaches

**Figure 1:**
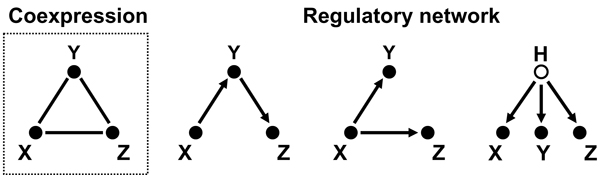
**Different mechanisms can explain coexpression**. The left plot in the dashed box shows three coexpressed genes forming a clique in the coexpression graph. The other three plots show possible regulatory relationships that can explain coexpression: The genes could be regulated in a cascade (left), or one regulates both others (middle), or there is a common "hidden" regulator (right), which is not part of the model.

Even high similarity of expression tells us little about the underlying biological mechanisms. Coexpression networks include regulatory relationships, but we cannot distinguish direct from indirect dependencies based on the similarity of expression patterns. Fig. 1 exemplifies this problem on a small set of three highly coexpressed genes, which form a clique (a completely connected subgraph) in a coexpression network. The figure shows that several regulatory mechanism can explain this observation, and from coexpression data alone we have no way of choosing between them. There are two possible solutions. Functional genomics has a long tradition of perturbing the natural state of a cell and inferring a gene's function from the observed effects. These interventions allow us to distinguish between the three models in Fig. 1, because each model results in different predictions of effects, which can be compared to those obtained in experiments. For example, perturbing gene *Y *in the cascade *X *→ *Y *→ *Z *will only have an effect on gene *Z *but none on gene *X*. In the case where *Y *regulates both *X *and *Z*, perturbing it will result in changes at both regulatees. In the last case, where all three genes are regulated by a hidden regulator, perturbing one of them will not lead to changes at the other two. Methods formalizing these considerations are covered in the second part of this review. In the absence of perturbation data statistical methods may be used to find which of the possibilities is most likely. The theoretical background is the concept of *conditional independence*.

#### Conditional independence

Let *X*, *Y*, *Z *be random variables with joint distribution *P*. We say that *X is conditionally independent of Y given Z *(and write *X *⊥ *Y *| *Z*) if and only if

*P*(*X *= *x*, *Y *= *y *| *Z *= *z*) = *P*(*X *= *x *| *Z *= *z*)·*P*(*Y *= *y *| *Z *= *z*).

This is the same as saying

*P*(*X *= *x *| *Y *= *y*, *Z *= *z*) = *P*(*X *= *x *| *Z *= *z*)

and is a direct generalization of the independence condition for *X *and *Y*,

*P*(*X *= *x*, *Y *= *y*) = *P*(*X *= *x*)·*P*(*Y *= *y*).

The same definitions hold if conditioning is not on a single variable *Z *but on a set of variables ***Z***. For an interpretation, we can think of random variables as abstract pieces of knowledge obtained from, say, reading books [15]. Then *X *⊥ *Y *| *Z *means: "Knowing *Z*, reading *Y *is irrelevant for reading *X*"; or in other words: "If I already know *Z*, then *Y *offers me no new information to understand *X*." Variable *Z *can explain the correlation between *X *and *Y*.

The statistical models we discuss in the following all build on the concept of conditional independence. Instead of retrieving sets of coexpressed genes – as coexpression networks do – they try to recover the regulatory relationships between genes. To decide on an edge between *X *and *Y *in the graph, statistical models ask questions of the form "Is *X *independent of *Y *given ***Z***?", but differ with respect to what ***Z ***stands for: either all other variables except for *X *and *Y*, or single third variables, or any subset of all the other variables. Coexpression networks can be seen as the special case ***Z ***= ∅, which encodes marginal dependencies.

### Full conditional models

Full conditional models (also called Markov networks, undirected graphical models, or Markov random fields) ask: "Can the correlation observed between two genes be explained by *all other genes *in the model?" Nodes *i *and *j *are connected by an edge if and only if

*X*_*i *_⊥
 MathType@MTEF@5@5@+=feaafiart1ev1aaatCvAUfKttLearuWrP9MDH5MBPbIqV92AaeXatLxBI9gBaebbnrfifHhDYfgasaacH8akY=wiFfYdH8Gipec8Eeeu0xXdbba9frFj0=OqFfea0dXdd9vqai=hGuQ8kuc9pgc9s8qqaq=dirpe0xb9q8qiLsFr0=vr0=vr0dc8meaabaqaciaacaGaaeqabaqabeGadaaakeaacuGHLkIxgaGcaaaa@2E6E@*X*_*j *_| ***X***_rest_,

where "rest" denotes the set of all variables in *V *without *i *and *j*. Full conditional models become especially simple in a Gaussian setting. Assume that ***X ***~N (*μ*, Σ), where *μ*, is the mean vector and the covariance matrix Σ is invertible. Let *K *= Σ^-1 ^be the *concentration matrix *of the distribution (also called the *precision matrix*). The value -*k*_*ij*_/kiikjj
 MathType@MTEF@5@5@+=feaafiart1ev1aaatCvAUfKttLearuWrP9MDH5MBPbIqV92AaeXatLxBI9gBaebbnrfifHhDYfgasaacH8akY=wiFfYdH8Gipec8Eeeu0xXdbba9frFj0=OqFfea0dXdd9vqai=hGuQ8kuc9pgc9s8qqaq=dirpe0xb9q8qiLsFr0=vr0=vr0dc8meaabaqaciaacaGaaeqabaqabeGadaaakeaadaGcaaqaaiabdUgaRnaaBaaaleaacqWGPbqAcqWGPbqAaeqaaOGaem4AaS2aaSbaaSqaaiabdQgaQjabdQgaQbqabaaabeaaaaa@354C@ is called the *partial correlation coefficient *between genes *i *and *j *[15]. Then it holds for *i*, *j *∈ *V *with *i *≠ *j *that

*X*_*i *_⊥ *X*_*j *_| ***X***_rest _⇔ *k*_*ij *_= 0.

**Figure 2:**
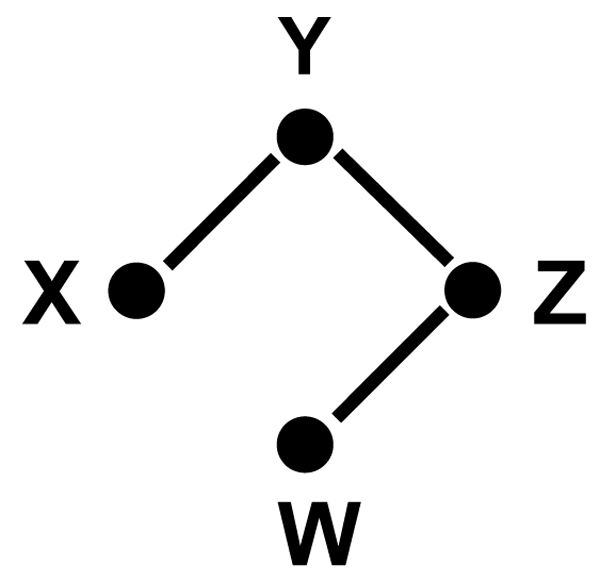
**A small Gaussian graphical model**. Example of a full conditional model. Missing edges between nodes indicate independencies of two genes given all the other genes in the model. We can read from the graph that *X *⊥ *W *| {*Y*, *Z*} and *Y *⊥ *W *| {*X*, *Z*} and *X *⊥ *Z |* {*Y*, *W*}.

This relation is used to define Gaussian graphical models (GGMs) [15,16]. A GGM is an undirected graph on vertex set *V*. Each vertex *i *∈ *V *corresponds with a random variable *X*_*i *_∈ ***X***. The edge set of a GGM is defined by non-zero partial correlations. Vertices *i *and *j *are adjacent if and only if *k*_*ij *_≠ 0. An example is shown in Fig. 2.

To estimate a GGM from data we need to know which elements of the precision matrix *K *are zero. This can be either done jointly for all edges, or by using non-exhaustive search algorithms like forward and backward search [15-17]. Alternatively, hypothesis testing-based model selection can be pursued by testing each edge separately for inclusion [18].

#### Comparison to correlation networks

Correlation graphs visualize the structure encoded in the correlation matrix Σ, which tells us about the similarity of expression profiles. In GGMs, we model using the precision matrix *K *= Σ^-1^, which shows correlation after correcting for the influence of all other genes. GGMs not only filter out all high correlations, which can be attributed to other genes, but may also draw attention to genes which are only very weakly correlated with a gene of interest, but highly related in terms of partial correlations in the context of the other neighboring genes in the GGM. These genes can be overlooked in correlation networks [19,20]. GGMs have a second clear advantage over correlation networks. Whether directly or indirectly, almost all genes will be correlated. Thus, the *correlation coefficient *is a weak criterion for dependence, but zero correlation is a strong indicator for independence. On the other hand, *partial correlation coefficients *usually vanish. They provide a strong measure of dependence and, correspondingly, only a weak criterion of independence [21].

#### Problems with GGMs

Full conditional relationships can only be accurately estimated if the number of samples *N *is relatively large compared to the number of variables *p*. If the number of genes to be analyzed exceeds the number of distinct expression measurements (that is, if *p *≫ *N*), the correlation matrix of expression profiles between genes does not have full rank and cannot be inverted [21]. The *p *≫ *N*-situation is true for almost all genomic applications of graphical models. Therefore, one must either improve the estimators of partial correlations or resort to a simpler model. The basic idea in all of these approaches is that biological data are high-dimensional but *sparse *in the sense that only a small number of genes will regulate one specific gene of interest.

Several papers suggest ways to estimate GGMs in a *p *≫ *N*-situation. Kishino and Waddell [22] propose gene selection by setting very low partial correlation coefficents to zero. As they state, the estimate still remains unstable. In one study, Schäfer and Strimmer [21] use bootstrap resampling together with the Moore-Penrose pseudoinverse and false discovery rate multiple testing, while in another [23], they discuss a linear shrinkage approach to regularization. Li and Gui [24] prospose a threshold gradient descent regularization procedure for estimating a sparse precision matrix.

#### Heuristic regression-based estimation

Full conditional independence models are closely related to a class of graphical models called dependency networks [25]. Dependency networks are built using sparse regression models to regress each gene *X*_*i *_onto the remaining genes ***X***_*V *\ *i*_. The genes, which predict the state of gene *X*_*i *_well, are connected to it by (directed) edges in the graph. In general, dependency networks may be inconsistent, *i.e*. the local regression models may not consistently specify a joint distribution over all genes. Thus, the resulting model is only an approximation of the true full conditional model. Still, dependency networks are widely used because of their flexibility and the computational advantage compared to structure learning in full conditional independence models. When learning dependency networks, a variety of sparse classification/regression techniques may be used to estimate the local distributions, including linear models with an *L*_1_-penalty on model parameters [20,26], classification trees [25,27], or sparse Bayesian regression [19,28]. We will see later that these approaches are very similar to the local regression models used in Bayesian networks. The difference is that in Bayesian networks an additional order of the variables is enforced.

### Low-order conditional independence

Full conditional models are hard to estimate if the number of samples is small compared to the number of genes. While the last section described different statistical techniques for *p *≫ *N*-situations, this section introduces the complementary approach. Instead of enhancing the estimation procedure, one can ask a simpler question: "Can the correlation between two genes be explained by a *single third gene*?" In contrast to GGMs, low-order conditional independence models condition not on the rest of the genes, but only on single third genes. An edge between vertices *i *and *j*(*i *≠ *j*) is drawn if the correlation coefficient *ρ*_*ij *_≠ 0 and no third gene can explain the correlation:

*X*_*i *_⊥
 MathType@MTEF@5@5@+=feaafiart1ev1aaatCvAUfKttLearuWrP9MDH5MBPbIqV92AaeXatLxBI9gBaebbnrfifHhDYfgasaacH8akY=wiFfYdH8Gipec8Eeeu0xXdbba9frFj0=OqFfea0dXdd9vqai=hGuQ8kuc9pgc9s8qqaq=dirpe0xb9q8qiLsFr0=vr0=vr0dc8meaabaqaciaacaGaaeqabaqabeGadaaakeaacuGHLkIxgaGcaaaa@2E6E@*X*_*j *_| *X*_*k *_for all *k *∈ *V*\{*i*, *j*}.

This general idea can be implemented in different ways: In a Gaussian setting, first order conditional independence models were proposed by several authors [29-32]. Testing for first order conditional independence involves only triples of genes at a time; thus, the problem for GGMs in high dimensions no longer exists. Wille *et al*. [30] use sparse Gaussian graphical modelling to identify modules of closely related genes and candidate genes for cross-talk between pathways in the Isoprenoid gene network in *Arabidopsis thaliana*.

In another approach, Margolin *et al*. [33] use conditional mutual information to test for first-order independence. The resulting method is called ARACNe and has been applied to expression profiles of human B cells [34]. The advantage of this approach is that the Gaussian assumption is dropped. However, this increased flexibility comes at a prize: ARACNe involves a number of computational approximations and Monte Carlo simulations, which could make the method unstable.

## Bayesian networks

In the last sections we have seen methods to build graphs from marginal dependencies (*X*_*i *_⊥
 MathType@MTEF@5@5@+=feaafiart1ev1aaatCvAUfKttLearuWrP9MDH5MBPbIqV92AaeXatLxBI9gBaebbnrfifHhDYfgasaacH8akY=wiFfYdH8Gipec8Eeeu0xXdbba9frFj0=OqFfea0dXdd9vqai=hGuQ8kuc9pgc9s8qqaq=dirpe0xb9q8qiLsFr0=vr0=vr0dc8meaabaqaciaacaGaaeqabaqabeGadaaakeaacuGHLkIxgaGcaaaa@2E6E@*X*_*j*_), full conditional dependencies (*X*_*i *_⊥
 MathType@MTEF@5@5@+=feaafiart1ev1aaatCvAUfKttLearuWrP9MDH5MBPbIqV92AaeXatLxBI9gBaebbnrfifHhDYfgasaacH8akY=wiFfYdH8Gipec8Eeeu0xXdbba9frFj0=OqFfea0dXdd9vqai=hGuQ8kuc9pgc9s8qqaq=dirpe0xb9q8qiLsFr0=vr0=vr0dc8meaabaqaciaacaGaaeqabaqabeGadaaakeaacuGHLkIxgaGcaaaa@2E6E@*X*_*j *_| ***X***_rest_), or first order dependencies (*X*_*i *_⊥
 MathType@MTEF@5@5@+=feaafiart1ev1aaatCvAUfKttLearuWrP9MDH5MBPbIqV92AaeXatLxBI9gBaebbnrfifHhDYfgasaacH8akY=wiFfYdH8Gipec8Eeeu0xXdbba9frFj0=OqFfea0dXdd9vqai=hGuQ8kuc9pgc9s8qqaq=dirpe0xb9q8qiLsFr0=vr0=vr0dc8meaabaqaciaacaGaaeqabaqabeGadaaakeaacuGHLkIxgaGcaaaa@2E6E@*X*_*j *_| *X*_*k *_for all *k *∈ rest). The logical next step is to ask for independencies *of all orders*. In the resulting graph, two vertices *i *and *j *are connected if *no subset *of the other variables can explain the correlation, that is, if

*X*_*i *_⊥
 MathType@MTEF@5@5@+=feaafiart1ev1aaatCvAUfKttLearuWrP9MDH5MBPbIqV92AaeXatLxBI9gBaebbnrfifHhDYfgasaacH8akY=wiFfYdH8Gipec8Eeeu0xXdbba9frFj0=OqFfea0dXdd9vqai=hGuQ8kuc9pgc9s8qqaq=dirpe0xb9q8qiLsFr0=vr0=vr0dc8meaabaqaciaacaGaaeqabaqabeGadaaakeaacuGHLkIxgaGcaaaa@2E6E@*X*_*j *_| ***X***_*S *_for all *S *⊆ *V*\{*i*, *j*}.

This includes testing marginal, first order and full conditional independencies. Thus, the number of edges will be smaller compared to the models in the previous sections. The graph encoding the above independence statements for all pairs of nodes is still undirected. It can be shown that knowing independences of all orders gives an even higher resolved representation of the correlation structure. The collection of independence statements already implies directions of some of the edges in the graph [35-37]. The resulting directed probabilistic model is called a *Bayesian network*.

### Definition of a Bayesian network

A (static) Bayesian network is a graphical representation of the dependency structure between the components of a random vector ***X***. The individual random variables are associated with the vertices of a directed acyclic graph (DAG) *G*, which describes the dependency structure. Each node is described by a local probability distribution (LPD) and the joint distribution *p*(***x***) over all nodes factors as

p(x)=∏v∈Vp(xv|xpa(v),θv),
 MathType@MTEF@5@5@+=feaafiart1ev1aaatCvAUfKttLearuWrP9MDH5MBPbIqV92AaeXatLxBI9gBaebbnrfifHhDYfgasaacH8akY=wiFfYdH8Gipec8Eeeu0xXdbba9frFj0=OqFfea0dXdd9vqai=hGuQ8kuc9pgc9s8qqaq=dirpe0xb9q8qiLsFr0=vr0=vr0dc8meaabaqaciaacaGaaeqabaqabeGadaaakeaacqWGWbaCcqGGOaakieWacqWF4baEcqGGPaqkcqGH9aqpdaqeqbqaaiabdchaWjabcIcaOiabdIha4naaBaaaleaacqWG2bGDaeqaaOGaeiiFaWNae8hEaG3aaSbaaSqaaiabdchaWjabdggaHjabcIcaOiabdAha2jabcMcaPaqabaGccqGGSaaliiGacqGF4oqCdaWgaaWcbaGaemODayhabeaakiabcMcaPiabcYcaSaWcbaGaemODayNaeyicI4SaemOvayfabeqdcqGHpis1aaaa@4CFA@

where *θ*_*v *_denotes the parametrization of the local distribution and ***x***_*pa*(*v*) _is the vector of parent states denoting the activity levels of a gene's regulators. The DAG structure implies an ordering of the variables. The parents of each node are those variables that render it independent of all other predecessors. The factorization of the joint distribution is the key property of Bayesian networks. It allows to segment the set of variables into families, which can be treated individually. This basic definition of Bayesian networks poses a number of further questions, which are addressed in the following: (1.) How do the local probability distributions *p*(*x*_*v *_| ***x***_*pa*(*v*)_, *θ*_*v*_) look like? (2.) How is conditional independence defined for DAGs? (3.) How can we learn a Bayesian network structure from data? (4.) Are there natural limits to structure learning?

### Local probability distributions (LPDs)

Bayesian network models differ with respect to assumptions about the local probability distributions *p*(*x*_*v *_| ***x***_*pa*(*v*)_, *θ*_*v*_) attached to each node *v *∈ *V*. There are two types of parametric LPDs used in practice: multinomial distributions for discrete nodes and Gaussian distributions (normal distributions) for continuous nodes. A discrete node with discrete parents follows a multinomial distribution parametrized by a set of probability vectors, one for each parent configuration. A continuous node with continuous parents follows a Gaussian distribution, where the mean is a linear combination of parent states. Conditional Gaussian (CG) networks are a combination of discrete and Gaussian networks. Continuous nodes follow a Gaussian distribution and are allowed discrete and continuous parents, while discrete nodes follow a multinomial distribution and are restricted to discrete parents. CG networks constitute the general class of graphical models studied in statistics [15].

Another kind of parametric LPDs are regression trees, as used by Segal *et al*. [38,39]. Each regression tree is a rooted binary tree with parents in the DAG as internal nodes and leaf nodes associated with univariate Gaussian distributions. The regression trees capture the local structure in the data, whereas the DAG describes the global structure [40,41].

Instead of the parametric approaches discussed so far, the relationship between parents and children in the DAG can also be modeled by non-parametric regression models [42-45]. The result is a non-linear continuous model. This is an advantage over multinomial or Gaussian Bayesian networks, which are either discrete or linear.

Bulashevska and Eils [46] constrain LPDs to noisy logic functions modelling activatory or inhibitory parent-child relations. This has the advantage of simplifying and regularizing the model, while at the same time making it easier to interpret.

Nachman *et al*. [47] use non-linear Michaelis-Mentens dynamics to model how the transcription rate of a gene depends on its regulators. This approach combines Bayesian networks with a biochemically realistic quantitative model of gene regulation.

### Conditional independence in directed graphs

**Figure 3:**
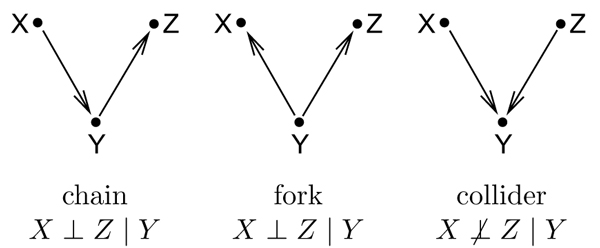
**Conditional indendence in directed graphs**. The three archetypal situations in the definition of d-separation. In the chain and the fork, conditioning on the middle node makes the others independent. In a collider, *X *and *Z *are marginally independent, but become dependent once *Y *is known.

In Fig. 2 we saw how to read off independence statements from a full conditional independence graph. How does this work in the case of Bayesian networks? The answer is given by the definition of *d-separation *[36] ("d" for directed), also called the *directed Global Markov condition *[15]. The three archetypical situations of d-separation (chain, fork, and collider) can be seen in Fig. 3. In a chain *X *→ *Y *→ *Z*, the middle node *Y *blocks the information flow between *X *and *Z *and thus it holds that *X *⊥ *Z *| *Y*. In a fork, where *X *and *Z *are both regulated by *Y*, knowing the state of the regulator renders the regulatees conditionally independent and thus again *X *⊥ *Z *| *Y*. The last case is more surprising: If *X *and *Z *are *independent *regulators with a common target *Y*, then the state of *Y *gives us information about *X *and *Z*. For example, imagine that *Y *is only expressed if only one of its regulators is active, then seeing *Y *expressed and *X *active implies *Z *being inactive. Thus, in the collider *X *→ *Y *← *Z *the middle node *Y *"unblocks" the path between *X *and *Z *and thus *X *⊥
 MathType@MTEF@5@5@+=feaafiart1ev1aaatCvAUfKttLearuWrP9MDH5MBPbIqV92AaeXatLxBI9gBaebbnrfifHhDYfgasaacH8akY=wiFfYdH8Gipec8Eeeu0xXdbba9frFj0=OqFfea0dXdd9vqai=hGuQ8kuc9pgc9s8qqaq=dirpe0xb9q8qiLsFr0=vr0=vr0dc8meaabaqaciaacaGaaeqabaqabeGadaaakeaacuGHLkIxgaGcaaaa@2E6E@*Z *| *Y*.

### Markov equivalence

Many Bayesian networks may represent the same statements of conditional independence. They are statistically undistinguishable and we call them *Markov equivalent*. All equivalent networks share the same underlying undirected graph (called the *skeleton*) but may differ in the direction of edges that are not part of a collider (also called a *v-structure*) [48]. Markov equivalence poses a theoretical limit on structure learning from data: even with infinitely many samples, we cannot resolve the structures in an equivalence class. In biological terms this means: even if we find two genes to be related it may not be clear which one is the regulator and which one is the regulatee. Without perturbation experiments this situation can not be further resolved.

### Acyclicity in a cyclic world

**Figure 4:**
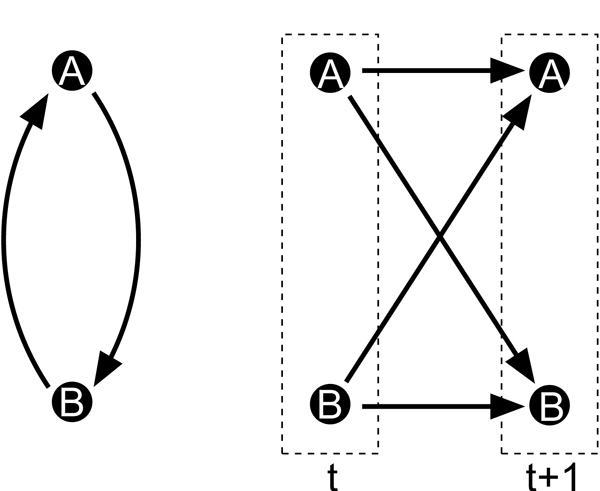
**Cycles unroll over time**. The cycle unrolls into an acyclic graph over different time slices.

Bayesian networks allow the highest resolution of correlation structure. Still, they suffer from a severe shortcoming: they are acyclic. With cycles, we cannot decompose the joint distribution as in the definition of Bayesian networks. Biological networks are all known to contain feedback loops and cycles [49]. Modeling the cell cycle with an acyclic model [50] can only be a preliminary step. One extension of Bayesian networks that encompasses cyclic structures is the factor graph network model of Gat-Viks *et. al*. [51]. A second way to address the cycle problem is by assuming that the system evolves over time. This is shown in Fig. 4. We no longer model a static random vector ***X ***but a time series ***X ***[1],..., ***X ***[*T*] of observing ***X ***at *T *timepoints. If we assume that *X*_*v *_at time *t *+ 1 can only have parents at time *t*, then cycles "unroll" and the resulting model is again acyclic and tractable: it is called a *Dynamic Bayesian network *(DBN) [52,53]. DBNs have found many applications in network reconstruction [54-56]. They are often augmented with hidden nodes [57], which can describe transcription factor activity [47] or any other kind of environmental or non-transcriptional effects in the cell [58-61]. In summary, DBNs provide one of the most flexible frameworks for modeling cellular networks.

### Score based structure learning

In correlation networks, GGMs and sparse GGMs we use statistical tests for each gene pair to decide whether the data support an edge or not. The number of tests to be done in these models is limited, even though it can be big in the case of sparse GGMs. For Bayesian networks we need to test independence of a gene pair for every subset of the other genes. This is called *constraint-based *learning of Bayesian networks [36,37]. For problems with more than a handful of variables testing becomes infeasible very quickly. In applications in computational biology the network structure is therefore mostly estimated by score based techniques. In the following we review maximum likelihood scores and Bayesian scores to evaluate model fit to data. Once the score is defined, model selection is posed as an optimization problem over the discrete space of possible model structures. Additional topics are how to fight overfitting and how to encode prior information.

#### Maximum likelihood

A straight-forward idea for model selection is to choose the DAG *G*, which allows the best fit to data ***D***. The best fit for a given DAG *G *is determined by maximizing the likelihood *p*(***D***|*G*, *θ*) as a function of *θ*, the parameters of the local probability distributions. A score for DAG *G *is then given by

scoreML(G)=max⁡θp(D|G,θ).
 MathType@MTEF@5@5@+=feaafiart1ev1aaatCvAUfKttLearuWrP9MDH5MBPbIqV92AaeXatLxBI9gBaebbnrfifHhDYfgasaacH8akY=wiFfYdH8Gipec8Eeeu0xXdbba9frFj0=OqFfea0dXdd9vqai=hGuQ8kuc9pgc9s8qqaq=dirpe0xb9q8qiLsFr0=vr0=vr0dc8meaabaqaciaacaGaaeqabaqabeGadaaakeaacqqGZbWCcqqGJbWycqqGVbWBcqqGYbGCcqqGLbqzdaWgaaWcbaGaemyta0KaemitaWeabeaakiabcIcaOiabdEeahjabcMcaPiabg2da9maaxababaGagiyBa0MaeiyyaeMaeiiEaGhaleaaiiGacqWF4oqCaeqaaOGaemiCaaNaeiikaGccbmGae4hraqKaeiiFaWNaem4raCKaeiilaWIae8hUdeNaeiykaKIaeiOla4caaa@4A35@

Unfortunately, the likelihood is not an appropriate score to decide between models since it tends to overfitting. Richer models with more edges will have a better likelihood than simpler ones, since the additional number of parameters allows a better fit to the data. A standard solution to this problem is to penalize the maximum likelihood score according to model complexity. An often used example of this general strategy is scoring with the Bayesian information criterion.

#### Bayesian information criterion (BIC)

Contrary to what the name suggests, the BIC score [62] is not a Bayesian score. It is a regularized maximum likelihood estimate, which controls overfitting by penalizing the maximal likelihood of the model with respect to the number of model parameters. It is defined as

scoreBIC(G)=max⁡θp(D|G,θ)−d2log⁡N,
 MathType@MTEF@5@5@+=feaafiart1ev1aaatCvAUfKttLearuWrP9MDH5MBPbIqV92AaeXatLxBI9gBaebbnrfifHhDYfgasaacH8akY=wiFfYdH8Gipec8Eeeu0xXdbba9frFj0=OqFfea0dXdd9vqai=hGuQ8kuc9pgc9s8qqaq=dirpe0xb9q8qiLsFr0=vr0=vr0dc8meaabaqaciaacaGaaeqabaqabeGadaaakeaacqqGZbWCcqqGJbWycqqGVbWBcqqGYbGCcqqGLbqzdaWgaaWcbaGaemOqaiKaemysaKKaem4qameabeaakiabcIcaOiabdEeahjabcMcaPiabg2da9maaxababaGagiyBa0MaeiyyaeMaeiiEaGhaleaaiiGacqWF4oqCaeqaaOGaemiCaaNaeiikaGccbmGae4hraqKaeiiFaWNaem4raCKaeiilaWIae8hUdeNaeiykaKIaeyOeI0YaaSaaaeaacqWGKbazaeaacqaIYaGmaaGagiiBaWMaei4Ba8Maei4zaCMaemOta4KaeiilaWcaaa@53A7@

where *d *is the number of parameters and the factor log *N *scales the penalty with respect to the likelihood. The BIC score can also be used to learn Bayesian networks with missing values or hidden variables. The likelihood has then to be maximized via the Expectation-Maximization (EM) algorithm. In such a scenario, the BIC score is used by Nachman *et al*. [47] to learn kinetic models of transcription factors and their targets. They treat protein activities and kinetic constants as hidden variables.

#### Bayesian scores

In most cases a full Bayesian approach is preferred over ML or BIC. In Bayesian structure learning we evaluate the posterior probability of model topology *G *given data ***D***:

scoreBayes=p(G|D)=p(D|G)⋅p(G)p(D).
 MathType@MTEF@5@5@+=feaafiart1ev1aaatCvAUfKttLearuWrP9MDH5MBPbIqV92AaeXatLxBI9gBaebbnrfifHhDYfgasaacH8akY=wiFfYdH8Gipec8Eeeu0xXdbba9frFj0=OqFfea0dXdd9vqai=hGuQ8kuc9pgc9s8qqaq=dirpe0xb9q8qiLsFr0=vr0=vr0dc8meaabaqaciaacaGaaeqabaqabeGadaaakeaacqqGZbWCcqqGJbWycqqGVbWBcqqGYbGCcqqGLbqzdaWgaaWcbaGaemOqaiKaemyyaeMaemyEaKNaemyzauMaem4Camhabeaakiabg2da9iabdchaWjabcIcaOiabdEeahjabcYha8Hqadiab=reaejabcMcaPiabg2da9maalaaabaGaemiCaaNaeiikaGIae8hraqKaeiiFaWNaem4raCKaeiykaKIaeyyXICTaemiCaaNaeiikaGIaem4raCKaeiykaKcabaGaemiCaaNaeiikaGIae8hraqKaeiykaKcaaiabc6caUaaa@5580@

The denominator *p*(***D***) is an average of data likelihoods over all possible models. This normalizing constant is the same for all models, and thus we do not need compute it to decide between competing models. The two main terms to consider in the Bayesian score are the prior over model structures, *p*(*G*), and the marginal likelihood *p*(***D***|*G*).

#### Marginal likelihood of network structure

The marginal likelihood *p*(***D***|*G*) is the key component of Bayesian scoring metrics. It equals the full model likelihood averaged over parameters of local probability distributions, that is,

p(D|G)=∫Θp(D|G,θ)p(θ|G)dΘ.
 MathType@MTEF@5@5@+=feaafiart1ev1aaatCvAUfKttLearuWrP9MDH5MBPbIqV92AaeXatLxBI9gBaebbnrfifHhDYfgasaacH8akY=wiFfYdH8Gipec8Eeeu0xXdbba9frFj0=OqFfea0dXdd9vqai=hGuQ8kuc9pgc9s8qqaq=dirpe0xb9q8qiLsFr0=vr0=vr0dc8meaabaqaciaacaGaaeqabaqabeGadaaakeaacqWGWbaCcqGGOaakieWacqWFebarcqGG8baFcqWGhbWrcqGGPaqkcqGH9aqpdaWdraqaaiabdchaWjabcIcaOiab=reaejabcYha8jabdEeahjabcYcaSGGaciab+H7aXjabcMcaPiabdchaWjabcIcaOiab+H7aXjabcYha8jabdEeahjabcMcaPiabbsgaKjabfI5arjabc6caUaWcbaGaeuiMdefabeqdcqGHRiI8aaaa@4C65@

Marginalization is the reason why the LPD parameters *θ *do not enter the definition of the posterior above. They are treated as *nuisance parameters *and have been integrated out. It is important to note that the LPD parameters were not maximized as it would be done in a maximum likelihood estimate or in a BIC score. Averaging instead of maximizing prevents the Bayesian score from overfitting.

Computation of marginal likelihood depends on the choice of local probability distributions and local priors in the Bayesian network model. To compute the marginal likelihood analytically, the prior *p*(*θ*|*G*) must fit to the likelihood *p*(***D***|*G*, *θ*). Statistically, this fit is called "conjugacy". A prior distribution is called *conjugate *to a likelihood, if the posterior is of the same distributional form as the prior [63]. If no conjugate prior is available, the marginal likelihood has to be approximated.

The marginal likelihood for discrete Bayesian networks was first computed by Cooper and Herskovits [64] and is further discussed by Heckerman *et al*. [65]. The conjugate prior for the multinomial distribution is the Dirichlet prior [63]. Assuming independence of the prior for each node and each parent configuration, the score decomposes into independent contributions for each family of nodes. Corresponding results exist for Gaussion networks using a Normal-Wishart prior [66]. The marginal likelihood again decomposes into node-wise contributions. Conditional Gaussian networks are a mix of discrete and Gaussian nodes [67]. The marginal likelihood decomposes into a Gaussian part and a discrete part.

For tree LPDs, the marginal likelihood at each node of the DAG further splits into independent components for each leaf of the local regression tree. Conjugate analysis and analytic results are possible using normal-gamma priors for each leaf node [40,41].

For non-parametrix LPDs, Boolean logic LPDs, and kinetic modelling LPDs, conjugate analysis and analytic computation of the marginal likelihood are not possible. Imoto *et al*. [42] use a Laplace approximation to approach the true marginal likelihood. Bulashevska and Eils [46] use Gibbs sampling to estimate the model posterior *p*(*G*|***D***) and the parameter posterior *p*(*θ*|***D***). Nachman *et al*. [47] use the BIC score for model selection.

#### Structure prior

Structure priors *p*(*G*) help to focus inference on reasonable models by including biological prior knowledge or integrating different data sources. Very often the biological prior knowledge can be encoded in a prior network, which is then to be refined by statistical structure learning. The first idea is to restrict the search space to a – conveniently defined – vicinity V
 MathType@MTEF@5@5@+=feaafiart1ev1aaatCvAUfKttLearuWrP9MDH5MBPbIqV92AaeXatLxBI9gBamrtHrhAL1wy0L2yHvtyaeHbnfgDOvwBHrxAJfwnaebbnrfifHhDYfgasaacH8akY=wiFfYdH8Gipec8Eeeu0xXdbba9frFj0=OqFfea0dXdd9vqai=hGuQ8kuc9pgc9s8qqaq=dirpe0xb9q8qiLsFr0=vr0=vr0dc8meaabaqaciaacaGaaeqabaWaaeGaeaaakeaaimaacqWFveVvaaa@384B@ (P
 MathType@MTEF@5@5@+=feaafiart1ev1aaatCvAUfKttLearuWrP9MDH5MBPbIqV92AaeXatLxBI9gBamrtHrhAL1wy0L2yHvtyaeHbnfgDOvwBHrxAJfwnaebbnrfifHhDYfgasaacH8akY=wiFfYdH8Gipec8Eeeu0xXdbba9frFj0=OqFfea0dXdd9vqai=hGuQ8kuc9pgc9s8qqaq=dirpe0xb9q8qiLsFr0=vr0=vr0dc8meaabaqaciaacaGaaeqabaWaaeGaeaaakeaacqqGqbauaaa@3786@) of the prior network P
 MathType@MTEF@5@5@+=feaafiart1ev1aaatCvAUfKttLearuWrP9MDH5MBPbIqV92AaeXatLxBI9gBamrtHrhAL1wy0L2yHvtyaeHbnfgDOvwBHrxAJfwnaebbnrfifHhDYfgasaacH8akY=wiFfYdH8Gipec8Eeeu0xXdbba9frFj0=OqFfea0dXdd9vqai=hGuQ8kuc9pgc9s8qqaq=dirpe0xb9q8qiLsFr0=vr0=vr0dc8meaabaqaciaacaGaaeqabaWaaeGaeaaakeaacqqGqbauaaa@3786@. All the DAGs in the restricted search space are considered equally likely. This can be interpreted as a rigid structure prior of the form

p(G)={1/|V(P)|if G∈V(P),0else.
 MathType@MTEF@5@5@+=feaafiart1ev1aaatCvAUfKttLearuWrP9MDH5MBPbIqV92AaeXatLxBI9gBaebbnrfifHhDYfgasaacH8akY=wiFfYdH8Gipec8Eeeu0xXdbba9frFj0=OqFfea0dXdd9vqai=hGuQ8kuc9pgc9s8qqaq=dirpe0xb9q8qiLsFr0=vr0=vr0dc8meaabaqaciaacaGaaeqabaqabeGadaaakeaacqWGWbaCcqGGOaakcqWGhbWrcqGGPaqkcqGH9aqpdaGabaqaauaabaqaciaaaeaacqaIXaqmcqGGVaWlcqGG8baFt0uy0HwzTfgDPnwy1egaryqtHrhAL1wy0L2yHvdaiqaacqWFveVvcqGGOaakcqqGqbaucqGGPaqkcqGG8baFaeaacqqGPbqAcqqGMbGzcqqGGaaicqWGhbWrcqGHiiIZcqWFveVvcqGGOaakcqqGqbaucqGGPaqkcqGGSaalaeaacqaIWaamaeaacqqGLbqzcqqGSbaBcqqGZbWCcqqGLbqzcqGGUaGlaaaacaGL7baaaaa@5942@

A smoother way to guarantee that DAGs similar to the prior network P
 MathType@MTEF@5@5@+=feaafiart1ev1aaatCvAUfKttLearuWrP9MDH5MBPbIqV92AaeXatLxBI9gBamrtHrhAL1wy0L2yHvtyaeHbnfgDOvwBHrxAJfwnaebbnrfifHhDYfgasaacH8akY=wiFfYdH8Gipec8Eeeu0xXdbba9frFj0=OqFfea0dXdd9vqai=hGuQ8kuc9pgc9s8qqaq=dirpe0xb9q8qiLsFr0=vr0=vr0dc8meaabaqaciaacaGaaeqabaWaaeGaeaaakeaacqqGqbauaaa@3786@ get higher prior probability is the following. We measure the confidence of edge (*v*, *w*) by a value 0 <*κ*_*vw *_≤ 1. A structure prior can then be defined proportional to a product of weights *κ*_*vw *_over all edges (*v*, *w*):

p(G)∝∏v,w∈Vκvw.
 MathType@MTEF@5@5@+=feaafiart1ev1aaatCvAUfKttLearuWrP9MDH5MBPbIqV92AaeXatLxBI9gBaebbnrfifHhDYfgasaacH8akY=wiFfYdH8Gipec8Eeeu0xXdbba9frFj0=OqFfea0dXdd9vqai=hGuQ8kuc9pgc9s8qqaq=dirpe0xb9q8qiLsFr0=vr0=vr0dc8meaabaqaciaacaGaaeqabaqabeGadaaakeaacqWGWbaCcqGGOaakcqWGhbWrcqGGPaqkcqGHDisTdaqeqbqaaGGaciab=P7aRnaaBaaaleaacqWG2bGDcqWG3bWDaeqaaaqaaiabdAha2jabcYcaSiabdEha3jabgIGiolabdAfawbqab0Gaey4dIunakiabc6caUaaa@40A9@

**Figure 5:**
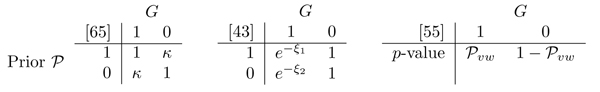
**An overview of structure priors**. Comparison of edge weights suggested by Heckerman *et al*. [65], Imoto *et al*. [43] and Bernard *et al*. [55]. Rows correspond to prior information. In the left two examples the prior can be described as binary, while on the right it is expressed as a *p*-value derived from a second data set. The entries in the table are the weights *κ*_*vw *_for each edge depending on whether *G *agrees with P
 MathType@MTEF@5@5@+=feaafiart1ev1aaatCvAUfKttLearuWrP9MDH5MBPbIqV92AaeXatLxBI9gBamrtHrhAL1wy0L2yHvtyaeHbnfgDOvwBHrxAJfwnaebbnrfifHhDYfgasaacH8akY=wiFfYdH8Gipec8Eeeu0xXdbba9frFj0=OqFfea0dXdd9vqai=hGuQ8kuc9pgc9s8qqaq=dirpe0xb9q8qiLsFr0=vr0=vr0dc8meaabaqaciaacaGaaeqabaWaaeGaeaaakeaacqqGqbauaaa@3786@ or not. The middle table holds *ξ*_1 _<*ξ*_2_, i.e. edges with motif evidence contribute more than edges without. The structure prior is then a product over the weights for all edges.

The normalization constant, which would be necessary to make the right-hand side a density, is the same for all models and can be ignored when computing relative posterior probabilities. What are smart choices of *κ*_*vw*_? There are several approaches suggested in the literature. Heckerman *et al*. [65] assume constant penalty *κ*_*vw *_≡ *κ *for all edges in which *G *and P
 MathType@MTEF@5@5@+=feaafiart1ev1aaatCvAUfKttLearuWrP9MDH5MBPbIqV92AaeXatLxBI9gBamrtHrhAL1wy0L2yHvtyaeHbnfgDOvwBHrxAJfwnaebbnrfifHhDYfgasaacH8akY=wiFfYdH8Gipec8Eeeu0xXdbba9frFj0=OqFfea0dXdd9vqai=hGuQ8kuc9pgc9s8qqaq=dirpe0xb9q8qiLsFr0=vr0=vr0dc8meaabaqaciaacaGaaeqabaWaaeGaeaaakeaacqqGqbauaaa@3786@ differ. Thus, *p*(*D*) ∝ *κ*^*ε *^where *ε *is the number of edges in which *G *differs from the prior DAG P
 MathType@MTEF@5@5@+=feaafiart1ev1aaatCvAUfKttLearuWrP9MDH5MBPbIqV92AaeXatLxBI9gBamrtHrhAL1wy0L2yHvtyaeHbnfgDOvwBHrxAJfwnaebbnrfifHhDYfgasaacH8akY=wiFfYdH8Gipec8Eeeu0xXdbba9frFj0=OqFfea0dXdd9vqai=hGuQ8kuc9pgc9s8qqaq=dirpe0xb9q8qiLsFr0=vr0=vr0dc8meaabaqaciaacaGaaeqabaWaaeGaeaaakeaacqqGqbauaaa@3786@. Another approach by Imoto *et al*. [43] and Tamada *et. al*. [45] uses a network prior in an iterative scheme. They construct a Bayesian network from microarray data, propose putative transcription factors from the network structure, and search for common motifs in the DNA sequences of children and grand-children of transcription factors. They then re-learn the network by penalizing edges without motif evidence harder than edges with motif evidence. Bernard *et al*. [55] define weights from *p*-values of binding location data. They assume that *p*-values follow an exponential distribution if the edge is present and a uniform distribution if it is not. By Bayes' rule they derive probabilities for an edge to be present given the *p*-values from the location data. The free parameter of the exponential distribution is then integrated out and the final probabilities P
 MathType@MTEF@5@5@+=feaafiart1ev1aaatCvAUfKttLearuWrP9MDH5MBPbIqV92AaeXatLxBI9gBamrtHrhAL1wy0L2yHvtyaeHbnfgDOvwBHrxAJfwnaebbnrfifHhDYfgasaacH8akY=wiFfYdH8Gipec8Eeeu0xXdbba9frFj0=OqFfea0dXdd9vqai=hGuQ8kuc9pgc9s8qqaq=dirpe0xb9q8qiLsFr0=vr0=vr0dc8meaabaqaciaacaGaaeqabaWaaeGaeaaakeaacqqGqbauaaa@3786@_*vw *_are used as weights in a structure prior. Fig. 5 shows a comparison of these three prior definitions. They can be organized by the weights *κ*_*vw *_they give for the presence or absence of an edge given prior information.

#### Discretization

In most applications the Bayesian score for discrete data is used. When learning gene regulatory networks from microarray data, it is necessary to preprocess the continuous gene expression values and discretize them. In general, discretization may be carried out for computational efficiency, or because background knowledge suggests that the underlying variables are indeed discrete. Discretizing continuous variables results in a loss of information, although it can also reduce noise, since discretized data can be more stable with respect to random variations of the mRNA measurements. Several methods to discretize microarray data have been proposed in the literature: Friedman *et al*. [50] discretize expression values into three categories, depending on whether the expression rate is significantly lower than, similar to, or greater than control, respectively. Pe'er *et al*. [68] introduce an adaptive discretization procedure. They model the expression level of a gene in different experiments as samples from a mixture of normal distributions, where each normal component corresponds to a specific state. They then use standard *k*-means clustering for inference. Hartemink *et al*. [69] use a discretization coalescence method, which incrementally reduces the number of discretization levels for each gene while preserving as much total mutual information between genes as possible. In the previous three approaches, expression levels were discretized before and independently of structure learning. Suboptimal discretization algorithms can lead to degraded network structure. To avoid this, Steck and Jaakkola [70] derive a scoring function to efficiently *jointly *optimize the discretization policy and the structure of the graphical model.

### Regularization

Regularization is a technique used in machine learning to ensure uniqueness of solution and to fight overfitting by constraining admissible models [14,71]. Regularization is always needed in *p *≫ *N*-situations. We already saw examples of regularization when Gaussian graphical models were adapted to the *p *≫ *N*-situation. Different methods were proposed for Bayesian networks. Steck and Jaakkola [72] show that scaling down the parameters of the Dirichlet prior used for the computation of the marginal likelihood leads to a strong regularization of the model structure and a sparse graph. Another way to regularize Bayesian networks is to constrain the local probability distributions. Bulashevska and Eils [46] suggest learning noisy logic gates instead of unconstrained multinomial LPDs. The drawback is that Bayesian conjugate analysis, which leads to the analytic solution of the marginal likelihood, is no longer possible and Gibbs sampling has to be applied. Module networks [38,39] constrain the number of parameters in the model by assuming that groups of genes (so called *modules*) share the same dependence on regulators. Learning module networks involves an iteration of assigning genes to modules and searching for dependencies between modules.

### Model selection and assessment

To search for the DAG with highest score is mathematically trivial: compute the score for every possible DAG and choose the one that achieves the highest value. What makes exhaustive search computationally infeasible in almost all applications is the huge number of DAGs. The number of DAGs on *n *edges is

an=∑k=1n(−1)k−1(nk)2k(n−k)an−k
 MathType@MTEF@5@5@+=feaafiart1ev1aaatCvAUfKttLearuWrP9MDH5MBPbIqV92AaeXatLxBI9gBaebbnrfifHhDYfgasaacH8akY=wiFfYdH8Gipec8Eeeu0xXdbba9frFj0=OqFfea0dXdd9vqai=hGuQ8kuc9pgc9s8qqaq=dirpe0xb9q8qiLsFr0=vr0=vr0dc8meaabaqaciaacaGaaeqabaqabeGadaaakeaacqWGHbqydaWgaaWcbaGaemOBa4gabeaakiabg2da9maaqahabaGaeiikaGIaeyOeI0IaeGymaeJaeiykaKYaaWbaaSqabeaacqWGRbWAcqGHsislcqaIXaqmaaaabaGaem4AaSMaeyypa0JaeGymaedabaGaemOBa4ganiabggHiLdGcdaqadaqaauaabeqaceaaaeaacqWGUbGBaeaacqWGRbWAaaaacaGLOaGaayzkaaGaeGOmaiZaaWbaaSqabeaacqWGRbWAcqGGOaakcqWGUbGBcqGHsislcqWGRbWAcqGGPaqkaaGccqWGHbqydaWgaaWcbaGaemOBa4MaeyOeI0Iaem4AaSgabeaaaaa@4FF8@

with *a*_0 _= 1 [73]. The number of DAGs increases explosively, as the first few steps in the recursion show: 1, 1, 3, 25, 543, 29 281, 3 781 503, 1 138 779 265. That means, we must use heuristic strategies to find high-scoring Bayesian networks without enumerating all possible DAGs.

#### Defining search space

First we need to decide how to describe models of interest. This defines the model space, in which we search for models describing the data well. To apply search heuristics we have to equip the search space with a neighborhood relation, that is, operators to move from one point of the search space to the next one. The most simple search space results from defining a neighborhood relation on DAGs. Two DAGs are neighbors if they differ by one edge, which is either missing in one of them or directed the other way round. Madigan *et al*. [74] and Chickering [75] restrict the search space to Markov equivalence classes of DAGs which uniquely describe a joint distribution. Thus, no time is lost in evaluating DAG models which are equivalent anyway. Friedman and Koller [76] search over orders of nodes rather than over network structures. They argue that the space of orders is smaller and more regular than the space of structures and has a much smoother posterior landscape.

#### Search heuristics

Most of the following search algorithms can be applied to all search spaces, even though they are usually applied to DAGs. They return a single best network. A simple and fast but still powerful method is *hill-climbing *by greedy search. First, choose a point in search space to start from, e.g. a random graph or the empty graph. Compute the posterior probability for all graphs in the neighborhood of the current graph and select the graph with highest score. Iterate until no graph in the neighborhood has a larger score than the current graph. This procedure finds local maxima of the Bayesian scoring metric. The K2-algorithm [64] is a variant of greedy search, which assumes that the order of nodes is known. Several approaches have been suggested to speed up model search. The *sparse candidate algorithm *[77] restricts the number of possible parents for each node by searching for pairs of nodes which are highly dependent. The *ideal parent algorithm *[47,78] constructs a parent profile perfectly explaining the child behaviour and uses it to guide parent selection and to restrict the search space. Peña *et al*. [79] grow Bayesian networks starting from a target gene of interest. They iteratively add to the Bayesian network parents and children of all the genes already included in it. The algorithm stops after a predefined number of steps and thus, intuitively, highlights the surrounding area of the seed gene without having to compute the complete Bayesian network over all genes. Friedman [80,81] introduces the *structural EM algorithm *to learn Bayesian networks in the presence of missing values or hidden variables. It is an extension of the Expectation-Maximization (EM) algorithm that performs structure search *inside *the EM procedure and shows improvements in terms of speed and accuracy.

#### Assessing uncertainty

The problem with optimal models is, as Edwards [16] puts it: "Any method (or statistician) that takes a complex multivariate dataset and, from it, claims to identify one true model, is both naive and misleading". The emphasis is on "one true model". A better strategy than choosing a single best model is exploring the whole posterior distribution. Unfortunately, direct sampling from the posterior is intractable. The most we know about the data distribution is the empirical distribution of observations in the dataset. A classical approach to assess variability in the data is bootstrapping [82]. The strategy is to sample with replacement from the observations in the data set to get a number of bootstrap datasets, and then learn a network on every bootstrap dataset. The relative frequency of network features in the resulting network structures can be used as a measure of reliability [50,68]. Bootstrap samples can contain multiple copies of identical data points. This implies strong statistical dependencies between variables when given a small dataset. As a consequence, the resulting network structure can be considerably biased towards denser graphs. Steck and Jaakkola [83] propose a correction for this bias. As another simple way to avoid the bootstrap-bias Steck and Jaakkola [70] use the *leave-k-out *method. Instead of resampling with replacement, *k *cases are left out of the dataset when estimating a model. Repeating this many times also gives an estimate of model variability.

Markov Chain Monte Carlo (MCMC) is a simulation technique which can be also used to sample from the posterior *p*(*G|**D***). Given a network structure, a new neighboring structure is proposed. This new structure is accepted with the Metropolis Hastings acceptance criterion [84]. The iteration of this procedure produces a Markov chain that under fairly general conditions converges in distribution to the true posterior. MCMC is used by Husmeier [85] to learn dynamic Bayesian networks. Madigan *et al*. [74] use MCMC over Markov equivalence classes and Friedman and Koller [76] over orders of nodes.

### Benchmarking

Graphical models visualize a multivariate dependency structure. They can only answer biological questions if they succeed in reliably and accurately reconstructing biologically relevant features of cellular networks. Unfortunately, rigorous assessment and benchmarking of methods are still rare. One of the first evaluation studies is by Smith *et al*. [86]. They sample data from a songbird's brain model and report excellent recovery success when learning a Bayesian network from it. Zak *et al*. [87] develop a realistic 10 gene network, where the biological processes at the different levels of transcription, translation and post-translational modifications were modeled with systems of differential equations. They show that linear and log-linear methods fail to recover the network structure. Husmeier [85] uses the same simulation network [87] to specify sensitivity and specificity of dynamic Bayesian networks. He demonstrates how the network inference performance varies with the training set size, the degree of inadequacy of prior assumptions, and the experimental sampling strategy. By analyzing ROC curves Husmeier can show fair performance of DBNs. Wimberly *et al*. [88] test 10 algorithms, including Boolean and Bayesian networks, on a simulation [89] of the genetic network of the sea urchin embryo [90]. They report that reconstruction is unreliable with all methods and that the performance of the better algorithms quickly degrades as simulations become more realistic. Basso *et al*. [34] show that their own method, ARACNe, compares favorably against static Bayesian networks on a simulated network with 19 nodes [54] – but only if the dataset includes several hundreds of observations. On the other hand, Hartemink [91] finds *dynamic *Bayesian networks to be even more accurate than ARACNe on datasets of the same size. In a recent comparative evaluation, Werhli *et al*. [92] find no significant differences between coexpression networks, Gaussian graphical models and Bayesian networks, if they are used on nonlinear simulated data and real data. They conclude that the higher computational cost of inferring Bayesian networks over GGMs and coexpression networks is often not justified.

All in all the results show severe limitations of graphical models for microarray data: They need a large sample size and capture only parts of biologically relevant networks. One reason for this shortcoming is that the models we discussed so far all use purely observational data, where the cellular network was not perturbed experimentally. In simulations [92-94] and on real data [3,92] it was shown that data from perturbation experiments greatly improve performance in network reconstruction. In Bayesian networks this improvement is especially pronounced [92]. Accordingly, the following section introduces methodology for learning from effects of interventions in a probabilistic framework.

## Learning from experimental interventions

Physicist Richard Feynman once said: "What I cannot create, I do not understand". This quote stresses the importance of action for understanding. A complex system is not understood solely by passive contemplation, it needs active manipulation by the researcher. In biology this fact is long known. Functional genomics has a long tradition of inferring the inner working of a cell by breaking it. "What I cannot break, I do not understand" is the credo of functional genomics research.

### Linking causes with effects

**Figure 6:**
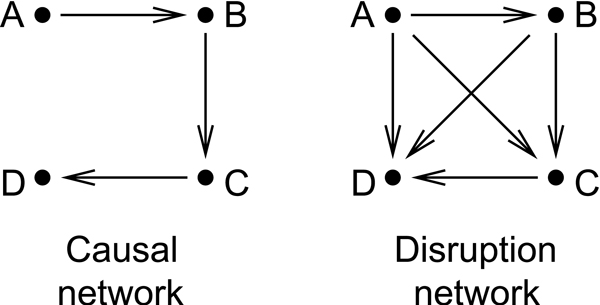
**Causal and discruption network**. From the causal network (left) it is easy to deduce how effects spread through the pathway (right). The harder problem is to deduce the causal pathway from observing effects of interventions (going from right to left).

Rung *et al*. [95] build a directed disruption graph by drawing an edge (*i,j*) if perturbing gene *i *results in a significant expression change at gene *j*. The authors focus on features of the disruption network that are robust over a range of significance cutoffs. Disruption networks do not distinguish between direct and indirect effects (and are in this sence similar to co-expression networks). Fig. 6 shows the difference between a causal network and a disruption network.

### Distinguishing direct from indirect effects

Disruption networks can be used as a starting point for further analysis. Wagner [96-98] uses graph-theoretic methods of *transitive reduction *[99,100] to find parsimonious subgraphs explaining all the effects in the disruption network. These methods are deterministic and do not take measurement noise into account. Wang and Cooper [101] describe a Bayesian generalization of the Wagner algorithm [98] yielding a distribution over possible causal relationships between genes.

### Boolean networks

A simple deterministic model of regulatory networks are Boolean networks: they are defined by a directed (and possibly cyclic) graph. For each node exists a boolean function relating parent states to the child state. Perturbations allow us to infer the structure and the logic of Boolean networks [102-104].

Unfortunately, deterministic models can not compensate for the noise inherent in biological systems. This is the reason why also for perturbation data probabilistic models are preferred.

#### Correlation

Rice *et al*. [105] build correlation graphs using knockout data. They assume that the data contain measurements of the unperturbed cell and several replicates of measurements for every gene knockout. For each gene *i*, they concatenate the wild-type data with the intervention data of this gene and compute on the joint data the correlation of gene *i *to all other genes. In the final graph, there is an arrow (*i, j*) if gene *j *was highly correlated to gene *i*. Since the correlation was computed on knockout data, the graph encodes causation and not only correlation. The big disadvantage of this method is the need for many (> 10) replicates of knockout experiments for *every *gene in the model, which is unrealistic for most real-world applications. Several regression methods make more efficient use of data.

#### Regression

Rogers and Girolami [28] use sparse Bayesian regression based on a Gaussian linear model to estimate a dependency network from knock-out data. They regress each gene onto all other genes by combining all the data corresponding to knockouts of genes other than the particular gene of interest. The measurements of the knockout gene are ignored when predicting this gene's expression from the other genes. We will see below that this strategy is the same as Pearl's *ideal interventions *used in Bayesian networks [36]. A prior on model parameters constrains most regression coefficients to zero and enforces a sparse solution. Non-zero regression coefficients are indicated by arrows in the regulation network. Other regression methods for network reconstruction are derived from a branch of engineering called *system identification *[106]. Functional relations between network components are inferred from measurements of system dynamics. Several papers [107-110] use multiple regression to model the response of genes and proteins to external perturbations.

### Ideal interventions

Pearl [36] proposes an idealized model of interventions in Bayesian networks. He assumes that an external manipulation completely controls the target node *v*. The influence of parent nodes *pa*(*v*) is cut and the LPD of *X*_*v *_degenerates to a point mass at the target state *x'*_*v*_, that is,

p(xv|xpa(v),θv)→interventionp(xv)={1if xv=x′v0else.
 MathType@MTEF@5@5@+=feaafiart1ev1aaatCvAUfKttLearuWrP9MDH5MBPbIqV92AaeXatLxBI9gBaebbnrfifHhDYfgasaacH8akY=wiFfYdH8Gipec8Eeeu0xXdbba9frFj0=OqFfea0dXdd9vqai=hGuQ8kuc9pgc9s8qqaq=dirpe0xb9q8qiLsFr0=vr0=vr0dc8meaabaqaciaacaGaaeqabaqabeGadaaakeaacqWGWbaCcqGGOaakcqWG4baEdaWgaaWcbaGaemODayhabeaakiabcYha8Hqabiab=Hha4naaBaaaleaacqWGWbaCcqWGHbqycqGGOaakcqWG2bGDcqGGPaqkcqGGSaalaeqaaGGacOGae4hUde3aaSbaaSqaaiabdAha2bqabaGccqGGPaqkdaGdKaWcbaGaeeyAaKMaeeOBa4MaeeiDaqNaeeyzauMaeeOCaiNaeeODayNaeeyzauMaeeOBa4MaeeiDaqNaeeyAaKMaee4Ba8MaeeOBa4gabeGccaGLsgcacqWGWbaCcqGGOaakcqWG4baEdaWgaaWcbaGaemODayhabeaakiabcMcaPiabg2da9maaceaabaqbaeaabiGaaaqaaiabigdaXaqaaiabbMgaPjabbAgaMjabbccaGiabdIha4naaBaaaleaacqWG2bGDaeqaaOGaeyypa0JafmiEaGNbauaadaWgaaWcbaGaemODayhabeaaaOqaaiabicdaWaqaaiabbwgaLjabbYgaSjabbohaZjabbwgaLjabc6caUaaaaiaawUhaaaaa@6DE3@

Ideal interventions are easily introduced into the computation of marginal likelihood. The key observation is that fixing a variable to a state tells us nothing about its "natural" behaviour. Cooper and Yoo [111] show that only cases in which a node was not fixed by an external manipulation enter into this node's contribution to the marginal likelihood. Ideal interventions were applied in Bayesian networks [3,68,94], factor graphs [51] and dependency networks [28]. In simulations [92-94] and on real data [3,92] it was found that interventions are critical for effective inference, particularly to establish directionality of the connections.

### Soft interventions

Pearl's model of ideal interventions contains a number of idealizations. The most important of these are that manipulations only affect single genes and that results can be controlled deterministically. The first assumption may not be true if there are compensatory effects involving other genes. The second assumption is also very limiting in realistic biological scenarios. Often the experimentalist lacks knowledge about the exact size of perturbation effects. To cope with this uncertainty, Markowetz *et al*. [112] introduced soft interventions as a generalization of ideal interventions. Variables are "pushed" in the direction of target states without fixing them. This idea is formalized in a Bayesian framework based on Conditional Gaussian networks.

### Physical network models

Yeang *et al*. [113] find the most likely annotated molecular interaction graph given a variety of data sources including gene-knockouts. Knock-out data is *functional *in nature and provides only indirect evidence about network structure. Yeang *et al*. associate each observed knock-out effect in the deletion mutant data with molecular cascades that could in principle explain the effect.

### Genetic interactions

Genetic interactions are defined by comparing the phenotypes of two single gene perturbations with the phenotype of the double gene perturbation. One example for a genetic interaction is epistasis [114]; it is defined as one gene masking the effect of another gene. Driessche *et al*. [115] use expression profiles as phenotypes and partly reconstruct a developmental pathway in *D. discoideum*. Another example of a genetic interaction is synthetic lethality, where two genes with a viable phenotype show a lethal phenotype in a double perturbation [116]. Wong *et al*. [117] propose a classification method to predict synthetic lethal interactions. Epistasis and synthetic lethality are just two examples of a broad range of possible genetic interactions. Drees *et al*. [118] define nine modes of genetic interactions for quantitative phenotypes that can be described by inequality constraints between the phenotypic values. They show that all modes of genetic interactions can be identified in agar-invasion phenotypes of mutant yeast.

### Nested effects models

A key obstacle to inferring genetic networks from perturbation screens is that phenotypic profiles generally offer only indirect information on how genes interact. Cell morphology or sensitivity to stresses are global features of the cell, which are hard to relate directly to the genes contributing to them. Gene expression phenotypes also offer only an indirect view of pathway structure due to the high number of non-transcriptional regulatory events like protein modifications. For example, when silencing a kinase we might not be able to observe changes in the activation states of other proteins involved in the pathway; the only information we may get is that genes downstream of the pathway show expression changes. Thus, phenotypic profiles may provide only indirect information about information flow and pathway structure.

**Figure 7:**
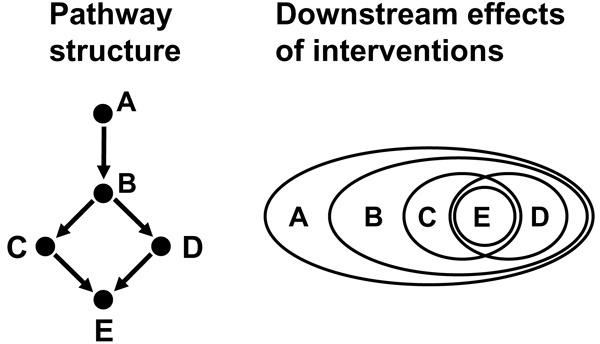
**Nested effects models**. Markowetz *et al*. [119] introduce a probabilistic model to infer a pathway structure (left) from the observed downstream effects of interventions (right). The model predicts that genes high up in the pathway (like ***A***) will have a broader set of effects than genes more downstream (like ***B ***to ***E***). The branching in the pathway below ***B ***corresponds to (partly) disjoint effect subsets for ***C ***and ***D***. The intersection of effect sets for ***C ***and ***D ***can be attributed to ***E ***and is explained by the collider at ***E ***in the pathway.

A recent approach [119] especially designed to learning from indirect information and high-dimensional phenotypes are *Nested Effects Models *that reconstruct features of the internal organization of the cell from the nested structure of observed perturbation effects. Perturbing some genes may have an influence on a global process, while perturbing others affects sub-processes of it. Imagine, for example, a signaling pathway activating several transcription factors. Blocking the entire pathway will affect all targets of the transcription factors, while perturbing a single downstream transcription factor will only affect its direct targets, which are a subset of the phenotype obtained by blocking the complete pathway. Figure 7 shows a schematic plot of how the position of perturbed genes in a pathway corresponds to a nested structure of observed effects. Markowetz *et al*. [119] demonstrate the power of Nested Effects Models in the controlled setting of simulation studies and explain its practical use in the context of an RNAi data set investigating the response to microbial challenge in *Drosophila melanogaster*.

### Experimental design

Statistical analysis of high-throughput data sets aims at generating *hypotheses *about the functions and regulatory roles of genes and proteins. Small-scale traditional experimental techniques are needed to verify the statistical predictions and inferred pathways. Experimental design or *active learning *deals with deciding which interventions to perform to discriminate optimally between alternative models. For reconstruction of regulatory networks, a number of methods have been proposed in different frameworks: for Bayesian networks [120,121], physical network models [122], Boolean networks [102], and dynamical modeling [123].

## Summary and Outlook

**Figure 8:**
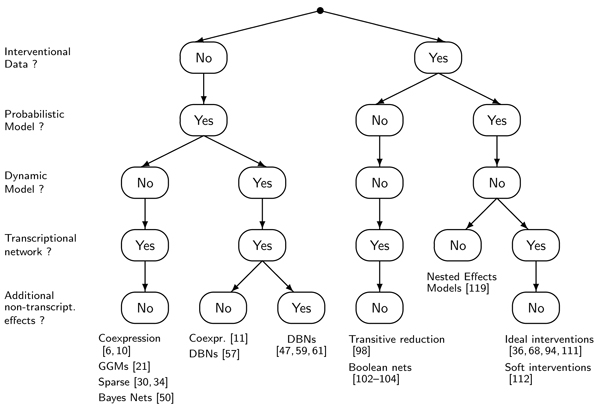
**A guide to the literature on network reconstruction**. Methodological similar approaches are clustered together by a decision tree built on five basic questions: Do the data contain knock-out or knock-down samples? Is the model deterministic or probabilistic? Does the model account for changes over time or is it static? Does the model focus on transcriptional gene regulation networks? And if so, does the model take additional non-transcriptional effects into account? In the leaf nodes of the tree, a few representative references are shown.

Fig. 8 organizes the network reconstruction methods we discussed in this review with respect to a few basic questions: Does the data include gene knockout or knockdown experiments? If not, we call it *purely observational data*; if yes, we call it *interventional data*. Is the model probabilistic or deterministic? Does the model allow for changes over time? If yes, we call it *dynamic*, otherwise *static*. Does the model describe transcriptional regulatory networks? And if so, are additional non-transcriptional effects taken into account? In the leaf nodes of the decision tree modeling techniques fall together that are methodologically similar. Fig. 8 shows representative examples. Some branches in the tree are missing, where we found no research on a given approach.

### The need for a holistic view

The internal organization of the cell comprises many layers. The *genome *refers to the collection of information stored in the DNA, while the *transcriptome *includes all gene transcripts. On the next level the *proteome *covers the set of all proteins. The *metabolome *contains small molecules – sugars, salts, amino acids, and nucleotides – that participate in metabolic reactions required for the maintenance and normal function of a cell. Results of internal reactions are features of the cell like growth or viability, which can be taken as *phenotypes *to study gene function. To understand the complexity of living cells future research will need to build models including all these layers. Statistical inference on parts of the system will not provide the mechanistic insights functional genomics is seeking for. Recent research concentrates on combining information from genome, transcriptome and proteome, *e.g*. for reconstructing signaling pathways [113,124] and networks of functionally related proteins [8,9,125]. This is a necessary step in the right direction. However, these models will still be fragmentary if they do not include and predict phenotypical changes of interventions perturbing the normal course of action in the cell. We will only understand what we can break.

## Further reading

There are a number of other reviews and method comparisons available: some focus on applications and biological interpretations [126-128], and some concentrate on methodology [129-137]. Even more sources can be found in a regularly updated bibliography [138].
